# An equilibrium for phenotypic variance in fluctuating environments owing to epigenetics

**DOI:** 10.1098/rsif.2011.0390

**Published:** 2011-08-17

**Authors:** Oana Carja, Marcus W. Feldman

**Affiliations:** Department of Biology, Stanford University, Stanford, CA 94305, USA

**Keywords:** epigenetics, epigenetic variation, phenotypic variation, fluctuating environments, evolution

## Abstract

The connection between random environments and genetic and phenotypic variability has been a major focus in the population genetic literature. By providing differential access to the underlying genetic information, epigenetic variation could play an important role in the interaction between environmental and phenotypic variation. Using simulation, we model epigenetic plasticity during development by investigating the dynamics of genetic regulators of the epigenetic machinery that change the variance of the phenotype, while having no effect on the phenotype's mean. Previous studies have found that increased phenotypic variance is selected for if the environment is fluctuating. Here, we find that when a variance-increasing allele achieves a sufficiently high frequency, it can be out-competed by a variance-reducing allele, with the consequence that the population evolves to an equilibrium phenotypic variability. This equilibrium is shown to be robust to different initial conditions, but to depend heavily on parameters of the model, such as the mutation rate, the fitness landscape and the nature of the environmental fluctuation. Indeed, if there is no mutation at the genes controlling the variance of the phenotype, reduction of this variance is favoured.

## 1. Introduction

Since the beginning of quantitative genetics [1–3], phenotypic variation has been understood to involve contributions from both genetic and environmental variation. The ability of organisms to phenotypically respond to environmental fluctuations has been recognized as a powerful adaptation [4–10]. Organisms have evolved an enormous variety of tactics that enable them to cope with environmental changes: some use behavioural or physiological modifications that leave no permanent trace in the genes of their descendants (traits acquired or learned during the lifetime of an individual; see, for example, [11–14]), while others respond to environmental change through the creation of diversity among their offspring, diversity that can either be genetic or non-genetic [15–18].

Starting with the early work of Haldane & Jayakar [19], Levins [20], Kimura [21], Ewens [22] and Felsenstein [23], quantitative connections between environmental variance, genetic variance and phenotypic variance have been an important component of evolutionary analysis. Dempster [24] introduced a model in which temporal fluctuations in reproductive success of competing genotypes favour the genotype with the highest mean reproductive rate. Jablonka *et al.* [25] found that carry-over effects (the persistence of a particular phenotype for one or more generations despite a change in the environmental conditions that first induced the phenotype) can provide an advantage in stochastic environments. Studies of the evolution of phenotypic plasticity (the capacity of a single genotype to result in different phenotypes that correlate with environmental variability) [26–29] suggest that increased plasticity has an advantageous effect in a non-stationary environment, allowing individuals to acclimate to rapid changes that cannot be tracked by the normally slow evolutionary process [30–32]. A different class of models has addressed the evolution of stochastic switching. Under stochastic environmental fluctuations, individual cells may switch among a number of different heritable phenotypes and this has been recognized as a possible case of bet-hedging [16–18,33–36]. These studies suggest that populations of cells tune these switching rates to the rate of the environmental fluctuations; that is, fast-switchers out-grow slow-switchers when the environment fluctuates rapidly [18,34]. These models suggest that increased phenotypic heterogeneity enhances the fitness of a population under an appropriately changing environment, because favoured phenotypes exist under each environmental condition.

Superimposed on the DNA is a layer of heritable epigenetic information that researchers have recently begun to read and understand. This epigenetic information is the result of chemical modifications to cytosine bases and/or to the histone proteins that package the genome. By regulating chromatin structure and DNA accessibility, these chemical changes influence how genes are expressed across a diverse array of developmental stages, tissue types, disease states and abiotic environments [37–41]. Epigenetic variation contributes to phenotypic variance without altering the genotype, by allowing the same structural genetic information to yield multiple cell types in different life cycle stages. It may also be responsible for potential alternative developmental pathways in an organism based on its own and its ancestors' environments. Therefore, understanding the role of epigenes in phenotypic variability might provide new insights into patterns of diversity in fluctuating environments.

Phenotypic variability mediated by epigenetic mechanisms was investigated in a recent simulation analysis [42] of genetic variants that do not change the mean phenotype, but do affect the variance of the phenotype. This model was intended to represent epigenetic plasticity during development, for example, by DNA methylation patterns that affect stochastic phenotypic variation through epigenetic mediators. It was inspired by the dietary modifications of DNA methylation of the *Agouti* gene, and methylation of the *Axin*-fused allele in kinked tail mice [43], which demonstrate how epigenetic differences can result in very diverse phenotypes among genetically identical individuals. The model differs from previous transgenerational epigenetic models [44–46], and from other stochastic-switching models because it focuses on the evolution of genes that control the expression of statistical variance in the phenotype without affecting the mean phenotype.

Some previous attempts to incorporate epigenetics into evolutionary models have focused on neo-Lamarckian inheritance, allowing for the limited inheritance of acquired characteristics [44], such as culturally transmitted traits [47,48]. Although these may be relevant in some cases, many epigenetic responses are determined and controlled by DNA-encoded genes (such as chromatin remodelling genes or genes that affect or detect DNA methylation) and, from an evolutionary perspective, epigenetic variation is, for the most part, likely to be subservient to the evolving DNA sequence. That is, the machinery of epigenetic modifications (e.g. DNA methyltransferases and histone deacetylases) is ultimately encoded by the DNA sequence, and whether a particular structural gene is subject to a particular epigenetic modification will be partly dependent on the properties of the DNA itself. Moreover, the rate of structural DNA mutation is much smaller than the mutation rate for epialleles, which are less stable [49].

In their model of stochastic epigenetic variation under fluctuating environments, Feinberg & Irizarry [42] provide two experimental results as proof of principle for the existence of genes that do not change the mean phenotype, but do change the variability of the phenotype. Their first experiment identified highly variable DNA-methylated regions in mouse and human liver and mouse brain associated with development and morphogenesis, thus supporting the concept of stochastic epigenetic variation. Their second example, the loss or gain of CpG dinucleotides, supports the existence of heritable genetic mechanisms (i.e. the underlying DNA sequence) that control methylation and as a consequence have an effect on the variability of the phenotype, through epigenetic variation. Using simulations to model the evolution of genes that control phenotypic variance, the authors find that, in a changing environment, the genetically inherited propensity for phenotypic variability substantially increases fitness. In this paper, we explore the model of Feinberg and Irizarry in more detail and find that their results hold only in a limited parameter range and only in the initial generations of the population's evolution.

We first replicate the simulations in Feinberg & Irizarry [42] and find that, in a fluctuating environment, if the initial phenotypic variability is small, then the phenotypic variance of the population does indeed increase initially; that is, for about 1000 generations. After a longer time, however, we observe that the population variance of the phenotype reaches an equilibrium that depends on the parameters of the model, but is robust to initial conditions.

We then use an explicit population genetic model with a modifier gene that determines the extent of variation of an individual's phenotype to explore the conditions under which an increase in phenotypic variance is adaptive and is selected for in a non-stationary environment. We find an equilibrium for the frequencies of the alleles at the locus controlling the phenotypic variability and study the properties of this equilibrium distribution by looking at a range of different model parameters and their effect on this equilibrium. We find that phenotypic variability does increase in populations undergoing rapid environmental change, but this effect disappears in environments that change very slowly. We also find that increased phenotypic variance seems to be favoured in populations that are already adapted to their environments as it may increase the populations' exploration of the phenotypic space. By contrast, the response to an environment that is particularly deleterious seems to be a decrease in phenotypic variability, as the fitness advantage of a beneficial phenotype does not overcome the costs of a deleterious one. We also find that this equilibrium depends strongly on the mutation rate at the modifier locus: if there is no mutation at the locus that affects phenotypic variability, an allele that increases the variance of an individual's phenotype will eventually be lost.

## 2. Results

### 2.1. The general model

The stochastic model we present below is based on that of Feinberg & Irizarry [42]. Consider a haploid population of fixed size, *N* = 10 000. Each individual in the population is defined by *N*_1_ genes (e.g. single nucleotide polymorphisms) that control mean phenotype and *N*_2_ genes that control the variability of its phenotype. We will denote by ***X*** the set of genes that determine mean phenotype and by ***M*** the set of genes that control the variance of an individual's phenotype. To incorporate the possibility that a gene is not expressed in the phenotype, ***X*** and ***M*** are assumed to be vectors with elements 0 or 1, with 1 denoting expression. Therefore, for each individual *i*, ***X*** and ***M*** can be represented as vectors of elements 0, 1 of size *N*_1_ and *N*_2_, respectively:
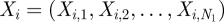
and
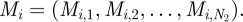
The phenotype *Y*_*i*_ of an individual with genotype (*X*_*i*_, *M*_*i*_) is given by
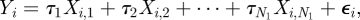
where the vector *τ* = (*τ*_1_, *τ*_2_, … , *τ*_*N*_1__) records the expected effects on the phenotype *Y*_*i*_ from the loci (*X*_*i*,1_, *X*_*i*,2_, … , *X*_*i*,*N*_1__) and *ε*_*i*_ represents the variation not explained by *X*_*i*_, which is added to the phenotype, with its variance determined by the genes *M*_*i*_:
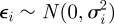
and
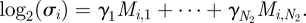
Here *γ* = (*γ*_1_, *γ*_2_, … , *γ*_*N*_2__) is the analogue of *τ*, on a log scale, and determines the effect of the (*M*_*i*,1_, *M*_*i*,2_, …, *M*_*i*,*N*_2__) loci on the variance of phenotype *Y*_*i*_.

We assume there are two different environments, *e*_1_ and *e*_2_. Given an individual's phenotype *Y*_*i*_, its probability of survival, i.e. fitness, in each of the two environments is computed as follows:
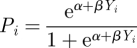
in environment *e*_1_ and
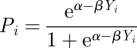
in environment *e*_2_ (general shapes of these functions are presented in figure 3*a*,*c*). Here, *α* and *β* are parameters that represent the baseline level of adaptation to the environment and the degree of difference between the two environments, respectively. If *β* is positive, positive phenotypes have a fitness advantage in environment *e*_1_, while negative phenotypes are better adapted to environment *e*_2_; the reverse is true for negative values of *β*.

To create the next generation, we sample *N* individuals from the current population, each individual having a probability of being selected that is proportional to its fitness. The three forces acting on the population are selection, mutation and random genetic drift owing to finite population size, in that order; we assume a constant mutation rate *μ* for all *N*_1_+*N*_2_ loci and no recombination.

**Figure 1.**
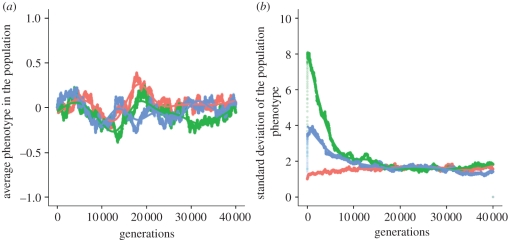
(*a*) Average and (*b*) standard deviation of phenotype *Y*_*i*_ in the population, as a function of generation time, over 40 000 generations. The parameters are: *α* =−4, *β* = 4, *μ* = 10^−4^, *τ* = (−1, −1, −1, −1, 1, 1, 1, 1), *γ* = (−1, −1, −1, −1, 1, 1, 1, 1)/2 and *N* = 10 000 individuals. The environment changes periodically every five generations and the simulation starts with an isogenic population at the *X* genes, *X*_*i*_ = (0, 0, 0, 0, 0, 0, 0, 0). There are three different initial conditions for the *M* genes: for every individual in the first generation, each one of its eight *M* genes is 0 with probabilities 0.9 (red curve), 0.5 (green curve) or 0 (blue curve). Each point represents the average across 100 different runs of the simulation. The curves represent a fit to the data using a generalized additive model with penalized cubic regression splines.

We first repeat the simulations in Feinberg & Irizarry [42], using the same parameter values and a wider range of initial conditions, but a longer evolutionary time. Set *N*_1_ = 8, *N*_2_ = 8, *α* =−4, *β* = 4, *μ* = 10^−4^, *τ* = (−1, −1, −1, −1, 1, 1, 1, 1) and *γ* = (−1, −1, −1, −1, 1, 1, 1, 1)/2. We assume that the environment changes periodically, every five generations. We start the simulation with an isogenic population at the ***X*** genes, *X*_*i*_ = (0, 0, 0, 0, 0, 0, 0, 0) and three different initial conditions for the ***M*** genes: for every individual in the first generation, each one of its eight ***M*** genes is 0 with probabilities 0.9, 0.5 or 0, respectively, for each of three different simulations presented in figure 1. We ran this simulation 100 times and averaged the results. Figure 1 shows the average and standard deviation of the phenotypes *Y*_*i*_ in the population, as a function of generation time, over 40 000 generations.

In figure 1*a*, the average phenotypic value of the population oscillates around a mean value of 0, as found by Feinberg & Irizarry [42]. Figure 1*b*, however, shows an interesting departure from their findings: while the standard deviation of the phenotypes *Y*_*i*_ in the population does increase initially, this increase eventually ceases and we observe the appearance of an equilibrium for the phenotypic variance. The existence of an equilibrium value for the phenotypic variance seems to be robust to the numbers of loci *N*_1_ and *N*_2_ used in the simulation: electronic supplementary material S1 presents a model with two loci controlling the mean phenotype (*X*_*i*,1_ and *X*_*i*,2_) and one modifier locus (*M*_*i*_) controlling the variance of the phenotype. We again observe a brief initial increase (for approx. 1000 generations), followed by approach to an equilibrium for the phenotypic variance in the population.

Sampling such large populations (*N* = 10 000) is computationally costly, and since the population size is large, the effects of genetic drift are likely to be negligible. We therefore propose that the results should be the same if we iterate the corresponding recursions for genotype frequencies in an infinite population model. In particular, we will examine the sensitivity of the above finding to changes in the model parameters.

### 2.2. A deterministic model

We construct a deterministic model of haploid individuals with *N*_1_ = 2 and *N*_2_ = 1; this is based on traditional population genetic theory models of modifier loci controlling a parameter of interest (see [50]). Thus, each individual is now defined by three genes: two genes *A*/*a* and *B*/*b* that control mean phenotype and one modifier gene *M*/*m* that controls its variance. We have eight possible haploid genotypes: *ABM*, *AbM*, *aBM*, *abM*, *ABm*, *Abm*, *aBm* and *abm*, with corresponding frequencies *x*_1_, *x*_2_, *x*_3_, *x*_4_, *x*_5_, *x*_6_, *x*_7_, *x*_8_. Following the previous model, we compute the phenotype values *Y*_*i*_ for these eight genotypes:
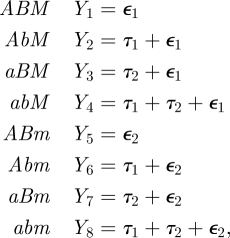
where *ε*_1_ ∼ *N*(0,*σ*_1_^2^ ) and *ε*_2_ ∼ *N*(0,*σ*_2_^2^). The *M*/*m* locus does not modify the mean phenotype; its sole effect is on the variance of the phenotype. In all of the following, we will set *τ*_1_ = −1 and *τ*_2_ = 1, thus ensuring that the alleles at the *A*/*a* and the *B*/*b* loci are, on average, symmetrically deleterious or beneficial in the two environments and any departures from the equilibrium genotype frequencies of 1/8 will be due to selection at the *M*/*m* modifier locus. (A more detailed explanation is in electronic supplementary material S1.) For phenotype *Y*_*i*_, the survival probability is
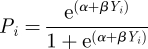
in environment *e*_1_ and
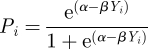
in environment *e*_2_. We study the dynamics of the frequencies of the modifier alleles over time, following the recursion equations for the frequencies of the eight genotypes in the next generation as a function of the genotype frequencies in the current generation; the complete recursion equations are presented in appendix A.

**Figure 2.**
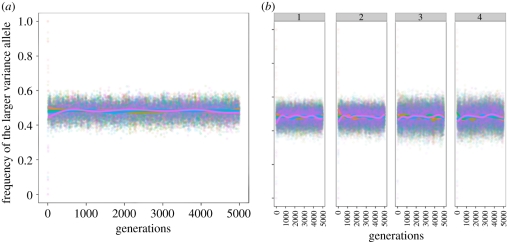
Robustness of the equilibrium to initial conditions at the *M*/*m* locus. The parameters are: *α* = (−4), *β* = 4, *μ* = 10^−4^, *σ*_1_ = 1, *σ*_2_ = 2, the environment changes every five generations and we start with different initial frequencies of the *M*/*m* alleles at the modifier locus. Each point represents the average across 100 different runs of the simulation. The plotted curves represent a fit to the data using a generalized additive model with penalized cubic regression splines. (*a*) Equal initial frequencies of the *A*/*a* and *B*/*b* alleles. (*b*) Panel 1 corresponds to starting frequencies for *A* and *B* of 0, panel 2 corresponds to initial frequencies of *A* and *B* of 0.5, panel 3 corresponds to initial frequencies of *A* allele of 0.9 and *B* allele of 0 and panel 4 corresponds to initial frequencies of alleles *A* and *B* of 0.1 and 0, respectively. (*a,b*) Initial frequencies of the larger variance allele: red curve, 1; light green curve, 0.9; dark green curve, 0.5; blue curve, 0.1; pink curve, 0.

In figure 2, we use the following parameter values: *α* = (−4), *β* = 4, *μ* = 10^−4^, *σ*_1_ = 1, *σ*_2_ = 2, and an environmental change every five generations. We average over 100 different runs of the simulation. The curves represent a fit to the data using a generalized additive model with penalized cubic regression splines. We show robustness of the results to other fitting techniques and the fitting curves with 95% confidence intervals in electronic supplementary material S5.

We first start with equal values of the alleles at the major loci *A*/*a* and *B*/*b* and different initial conditions for the *M*/*m* locus, as presented in figure 2*a*. We again observe an equilibrium for the frequencies of the alleles at the locus that controls phenotypic variance in the population. We next investigate how this equilibrium depends on the choice of the different model parameters. Of particular interest is the effect of the starting frequencies at the two major loci *A*/*a* and *B*/*b* on this equilibrium value. With the initial choice of *τ* = (−1,1), we guarantee that the loci modifying mean phenotype are not the drivers of the evolutionary process and the allelic frequencies are determined by selection operating at the *M*/*m* locus. We test this by performing simulations in which we change the starting allele frequencies at the *A*/*a* and *B*/*b* loci across a wide range of values. Four of these are shown in figure 2*b*: in panel 1, the frequencies of the *A* and *B* alleles start at zero; in panel 2, the frequencies of the *A* and *B* alleles start at 0.5; in panel 3, the frequency of the *A* allele is 0.9 and the frequency of the *B* allele is 0; in panel 4, the frequency of the *A* allele is 0.1 and the frequency of the *B* allele is 0. Figure 2*b* presents the results for different initial allele frequencies at the modifier locus, the other parameters being fixed at the following values: *α* = (−4), *β* = 4, *σ*_1_ = 1, *σ*_2_ = 2, *μ* = 10^−4^ and the environment changing every five generations. It is easy to see that different initial frequencies at the major loci *A*/*a* and *B*/*b* do not affect the equilibrium frequencies of alleles at the *M*/*m* locus.

We tested the existence of the equilibria over the whole range of parameters and present two more examples in electronic supplementary material S4.

**Figure 3.**
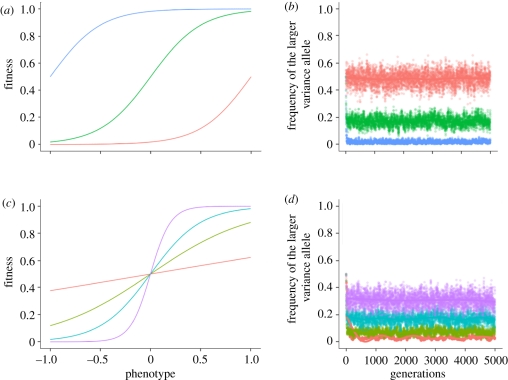
Effect of varying *α* and *β* on the equilibrium frequency at the *M*/*m* locus. (*a*) Fitness functions, varying the *α* parameter, with *β* = 4. (*b*) Effect of varying the *α* parameter on the equilibrium frequency at the *M*/*m* gene. The other parameters are *β* = 4, *μ* = 10^−4^, *σ*_1_ = 1, *σ*_2_ = 2 and the environment changes periodically every five generations. (*c*) Fitness functions, varying the *β* parameter, with *α* = 0. (*d*) Effect of varying the *β* parameter on the equilibrium frequency at the *M*/*m* locus. The other parameters are *α* = 0, *μ* = 10^−4^, *σ*_1_ = 1, *σ*_2_ = 2 and the environment changes periodically every five generations. In (*b*) and (*d*), each point represents the average across 100 different runs of the simulation. The plotted curves represent a fit to the data using a generalized additive model with penalized cubic regression splines. (*a*) *α*: red curve, −4; green curve, 0; blue curve, 4. (*b*) The *α* parameter: red curve, −4; green curve, 0; blue curve, 4. (*c*) *β*: red curve, 0.5; light green curve, 2; blue curve, 4; purple curve, 10. (*d*) The *β* parameter: red curve, 0.5; light green curve, 2; blue curve, 4; purple curve, 10.

We next examine the effect of *α* and *β* on the equilibrium frequency of the alleles at the *M*/*m* locus. It is first useful to visualize how a change in the parameter *α* affects the fitness function:
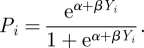
This is illustrated in figure 3*a*, where we fix *β* to be 4 and vary *α* to be − 4, 0 or 4. The plots correspond to the fitness function in environment *e*_1_, in which positive values of the phenotype *Y*_*i*_ have higher fitness and negative values have lower fitness; environment *e*_2_ is characterized by a change of sign of *β*, so the fitness function in *e*_2_ is simply the mirror image of that in *e*_1_. By varying *α* we can control how deleterious or beneficial the two environments are for the individuals in the population. Thus, *α* = 0 corresponds to symmetric environments, with positive phenotypes *Y*_*i*_ having a high fitness and negative phenotypes having very low fitness values. If *α* =−4, both environments are deleterious for both positive and negative phenotypes *Y*_*i*_. For *α* = 4, both environments are very favourable, one more so than the other.

To examine how the equilibrium frequency at the *M*/*m* gene responds to these different fitness functions, we iterated the population for the three different values of *α* mentioned above. The other parameters were kept constant: *β* = 4, *μ* = 10^−4^, *σ*_1_ = 1, *σ*_2_ = 2 and the environment changed every five generations. Figure 3*b* shows a clear effect of *α* on the equilibrium frequency of the larger variance allele *m*. For *α* =−4, in which one environment is extremely deleterious, we see that the phenotypic variability of the population oscillates around zero, meaning that, in order to escape the deleterious environment, the best strategy is to not explore the phenotypic space, but to base choices in the next environment on the current one. This makes sense, taking into account that the environment changes every five generations, so the probability of preserving the current environment is relatively high. For *α* = 0, corresponding to symmetric environmental fluctuations, the frequency of the larger variance allele oscillates around 0.2; again, larger phenotypic variance is not advantageous for the population. For *α* = 4, the equilibrium frequency of *m* oscillates around 0.5, a much larger value than in the previous two cases. Therefore, with one ideal environment and another beneficial environment, where the decrease in fitness from 1 is not very large, we observe a definite increase in phenotypic variability. This may be due to the fact that the benefits of exploring the phenotypic space outweigh the costs, and it is in these environments that strategies with even the slightest fitness advantage overcome competitors.

We next study the importance of the parameter *β* by fixing *α* = 0 and varying *β* to be 0.5, 2, 4 or 10. The fitness functions obtained are illustrated in figure 3*c* and differ mainly in the number of phenotypes that have fitness between the two extremes: 0 and 1. For *β* = 10, most phenotypes in the population have either very low or very high fitness, whereas for *β* = 0.5, most phenotypes *Y*_*i*_ have fitness values around 0.5. Keeping the other parameters constant at *α* = 0, *μ* = 10^−4^, *σ*_1_ = 1, *σ*_2_ = 2 and changing the environment every five generations, we plot the average allele frequencies at the *M*/*m* gene in figure 3*d*. We see that the equilibrium frequency of the larger variance allele decreases as *β* decreases. In environments where most phenotypes are around 0.5, there is no benefit to increased phenotypic variability. However, as we increase the fitness discrepancies between the two environments, increased phenotypic variability is favoured.

In the simulations below, we fix *α* =−4 and *β* = 4.

**Figure 4.**
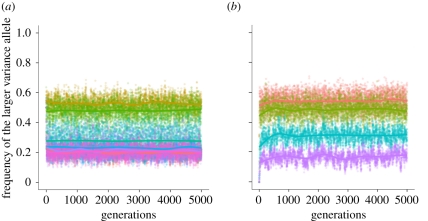
Effects of varying environmental periodicities and mutation rate *μ* on equilibrium frequencies at the *M*/*m* gene. (*a*) We vary the period of the environmental change (red curve, constant; brown curve, 1; light green curve, 5; dark green curve, 20; blue curve, 50; purple curve, 100; pink curve, 500). The parameters are *α* = (−4), *β* = 4, *μ* = 10^−4^, *σ*_1_ = 1, *σ*_2_ = 2. (*b*) We vary the mutation rate *μ* at the three loci and present results for four different mutation rates: 10^−2^ (red), 10^−4^ (green), 10^−8^ (blue), 10^−12^ (purple). The other parameters are *α* = (−4), *β* = 4, *μ* = 10^−4^, *σ*_1_ = 1, *σ*_2_ = 10 and the environment changes every five generations. Each point represents the average across 100 different runs of the simulation. The plotted curves represent a fit to the data using a generalized additive model with penalized cubic regression splines.

We would expect the population to respond differently to environments that do not change or change very slowly, compared with environments that are oscillating quickly, for example, every generation. In a highly variable environment, we would expect increased phenotypic variability to be selected for, allowing individuals to increase the range of accessible phenotypes in every generation. While this is the intuitive response to high uncertainty in environmental conditions, if the environment is constant, or changes very slowly, we would expect to see decreased phenotypic variability in the population, since selection would drive the population to an optimum, departures from which would be deleterious. To test this hypothesis, we first performed simulations for a range of environmental periods: constant environment and also periods 1, 5, 20, 50, 100 and 500. All other parameters were kept constant: *α* = −4, *β* = 4, *μ* = 10^−4^, *σ*_1_ = 1, *σ*_2_ = 2. Figure 4*a* shows that the population does indeed respond very differently to environments that change slowly, compared with fast-changing environments. For environments that change rapidly, the equilibrium frequency of the larger variance allele oscillates around 0.5, and is a decreasing function of the environmental periodicity. However, for environmental periodicities above 50 generations, the equilibrium oscillates around a value of 0.2. This suggests that organisms tend to respond to slowly changing environments in the same way they respond to a constant environment: if the environment in the next generation is likely to be preserved, then the population's best strategy is to decrease phenotypic variance, as variability may cause departure from the optimum. The robustness of the results to random environmental fluctuations, as presented in electronic supplementary material S2, is similar.

We next investigate the effect of the mutation rate at the three loci on the equilibrium frequency at the *M*/*m* gene. We performed simulations in which the range of mutation rates was 10^−2^, 10^−4^, 10^−8^ and 10^−12^. All other parameters were kept fixed at: *α* =−4, *β* = 4, *σ*_1_ = 1, *σ*_2_ = 2 and with an environmental change every five generations. Figure 4*b* shows a decrease in the equilibrium frequency of the larger variance allele with decreasing mutation rate at the three loci. It is interesting to note that the magnitude of this decrease is comparable to the decrease we observe when we ‘slow down’ the rate of environmental fluctuation. This decrease in the equilibrium frequency with decreasing mutation rate led us to ask what happens if mutation occurs at the loci that control mean phenotype, but not at the modifier locus, which controls the phenotypic variance.

With no mutation at the modifier locus, the genotype frequencies change according to the equations in appendix B. We used the same fixed parameters as before, *α* = −4, *β* = (4), *μ* = 10^−4^, *σ*_1_ = 1, *σ*_1_ = 1, *n* = 5, and recreated the conditions in figure 3, by varying the initial conditions at the modifier locus.

**Figure 5.**
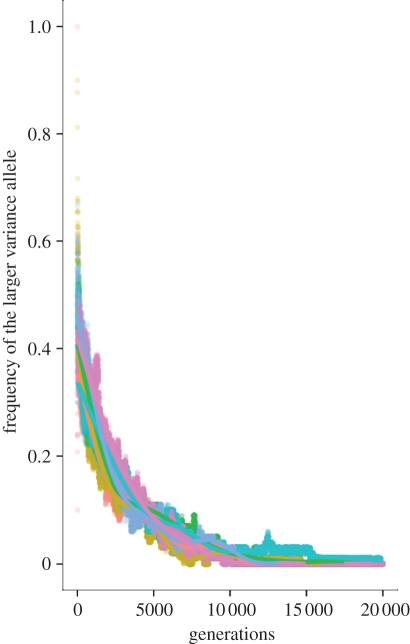
Simulations of the discrete model with no mutation at the locus controlling the phenotypic variance. We start with equal frequencies of the *A*/*a* and *B*/*b* alleles, and different initial frequencies at the *M*/*m* locus. The other parameters are *α* = (−4), *β* = 4, *μ* = 10^−4^, *σ*_1_ = 1, *σ*_2_ = 10 and the environment changes every five generations. Each point represents the average across 100 different runs of the simulation. The plotted curves represent a fit to the data using a generalized additive model with penalized cubic regression splines. Initial frequencies of the larger variance allele: red curve, 1; brown curve, 0.9; green curve, 0.6; blue curve, 0.5; purple curve, 0.4; pink curve, 0.1.

In figure 5, the frequency of the larger variance allele at the locus controlling the phenotypic variance is decreasing and approaches zero by generation 10 000. We have also performed extensive simulations varying all the other parameters of the model and in all simulations the larger variance allele always goes extinct by generation 10 000. The results suggest that when there is no mutation at the variance locus, the larger variance allele eventually disappears from the population; i.e. decreased phenotypic variability is favoured. This seems to imply that either there is no selection for increased phenotypic variance, or selection is very weak. In general, it is very difficult to distinguish between mutational variance and weak selection variance. The results in the other parameter regimes in the paper suggest the latter. We see that even though there is weak selection on the phenotypic variance, for a higher mutation rate, increased phenotypic variance can be selected for, as demonstrated by figures 3 and 4, for example.

## 3. Discussion

We have studied the impact of stochastic epigenetic variation on phenotypic variance in fluctuating environments. Motivated by the observation that there is significant and functionally important genetic variability in genes responsible for epigenetic control [39], we studied the dynamics of genes that are regulators of the epigenetic machinery. We modelled genes that do not change the mean phenotype of an individual, but control the variance of this phenotype. We have explored the conditions under which increased phenotypic variability is selected for, under a wide range of parameters and types of environmental fluctuations.

The model we presented is based on that of Feinberg & Irizarry [42] and population genetic theory of modifier alleles [50,51]. Their paper suggests that stochastic epigenetic variation is increased in fluctuating environments. We show that by 5000 generations the initial increase has reversed and a sharp decrease in the phenotypic variance of the population may occur. By about 10 000 generations, this variance has reached an equilibrium. This makes the conclusions of the model highly relevant for a large number of populations. Humans, for example, have a generation time of around 25 years. Therefore, the time to equilibrium would be 250 000 years, which is well under the time for the emergence and spread of *Homo sapiens neanderthalensis*. Thus, the approach to equilibrium variance is relevant for hominid evolution.

Our most important result is that there is an equilibrium for the alleles at the locus controlling the phenotypic variance of the individual, an equilibrium that may be very sensitive to changes in the different parameters of the model. This appears to be the first time such an equilibrium has been found. Previous studies of evolution in fluctuating environments suggest that increased phenotypic variance increases the fitness of a population in an appropriately changing environment and that a genetically inherited propensity for phenotypic variability substantially increases fitness and is selected for. The reasoning suggested is that variability promotes the existence of favoured phenotypes under each environmental condition. We have shown, however, that when a variance-increasing allele achieves a sufficiently high frequency, it can be out-competed by a variance-reducing allele, with the result that there is an optimum level of phenotypic variability. This optimum is shown to depend on the details of the model, namely the mutation rate, the fitness landscape and the nature of the environmental fluctuation.

The existence of such an equilibrium, robust to initial conditions, but sensitive to the other parameters of the model, seems to suggest that, in fact, the dynamics of these systems and the question of phenotypic variability are more complicated than previously thought. We have shown that whether increased phenotypic variability is favoured is very dependent on the characteristics of the studied population and the nature of the environmental fluctuations. If one of the environments is highly deleterious, decreased phenotypic variability is selected for, since there is a high cost (seen as a decrease in fitness) to departing from the optimum when the chance of being maladapted is high. By contrast, in beneficial environments, we observe selection for increased phenotypic variability—in this case, it is beneficial for individuals to explore the phenotypic space, since there are no major costs associated with this exploration. It is especially in these environments that organisms increase their phenotypic variance, taking advantage of even the slightest benefits available to them, as they compete with other moderately adapted individuals. One parameter that has a significant impact is the mutation rate at the modifier locus. In the absence of constant reintroduction of the allele that increases phenotypic variance, decreased phenotypic variance is selected for, irrespective of the other parameters of the model.

Our work tries to shed light on our understanding of the nature and relevance of phenotypic variation, especially in the context of changing environments. Is such variation available for adaptive change when a population undergoes a rapid change in environment and is therefore exposed to a new selection regime or is the variation simply a consequence of recurrent mutations being introduced in the population? Our results seem to imply the former, but further work is needed to understand the exact mechanisms that determine the existence of such equilibria and the exact dependencies of the stable levels of variation on the characteristics of the systems under study. Important progress on these issues could be made by integrating stochastic epigenetic variation into classic population genetic models of phenotypic variation. Epigenetic phenomena and their contribution to phenotypic variance have recently received considerable attention, both in theoretical [36,45,46,51,52] and experimental studies [43,53,53–56]. Nevertheless, our understanding of the molecular mechanisms of epigenomic regulation and the extent of its importance for phenotypic diversity is still far from complete.

The original definition of epigenetics by Waddington in [4]—the idea that phenotype arises from genotype through programmed change—is now central to developmental biology. The modern definition of epigenetics is information, other than the DNA sequence itself, that affects gene expression or function. There is extensive overlap between these two definitions: regulation of developmental processes by epigenetic phenomena may be central to development because different cell types maintain their differences during cell division even though their DNA sequences are essentially the same. Phenotypic change mediated by epigenetic change may, in some cases, be inherited across generations [46,57] and may lead to phenotypic heterogeneity among genetically identical individuals [58].

Epigenetic mechanisms appear to function primarily as genome defences, but may result in the maintenance of plasticity together with a degree of buffering of developmental programmes: breakdown of epigenetic buffering could potentially be deleterious for the organism and/or cause variation in rates of phenotypic evolution. Stochastic and environmentally induced epigenetic defects are also known to play a major role in cancer and ageing [58–60]. Two decades ago, cancer epigenetics was viewed with scepticism, but now it is widely accepted: there is compelling evidence that epigenetic marks, such as chromatin modification, can influence cellular phenotypes through the regulation of particular genes, without structural variation in these genes, and alterations in methylation, imprinting and chromatin are ubiquitous in cancer cells [61–63]. This also suggests that mutations in these genes involved in epigenetic control may have a major effect throughout development.

Population level models may prove valuable in showing potential ways in which epigenomic variation within populations may be related to phenotypic variation, and how patterns of epigenetic regulation may vary between individuals and genomic regions, as well as with the environment [64]. In order to develop such models, it is important to understand the sources of variation in epigenetic marks: they may be vertically transmitted, derive from parental environments, effects of ageing and of environment, stochastic events or genomic factors (for example, DNA sequence variants, expression differences in chromatin remodelling genes or genes that affect or detect DNA methylation).

In this paper, we have incorporated stochastic epigenetic variation into a population genetic model by studying the dynamics of genetic regulators of the epigenetic machinery that change the variance of the phenotype, while having no effect on the phenotype's mean. We found an equilibrium for the frequencies of the alleles at the locus controlling the phenotypic variability and showed that this equilibrium is robust to initial conditions, but depends on the details of the model, namely the mutation rate, the fitness landscape and the nature of the environmental fluctuation.

## Appendix A

Recursion equations for the frequencies of the eight genotypes under the infinite population model:

where  is the mean fitness of the population.

### Appendix B

Recursion equations for the frequencies of the eight genotypes under the infinite population model, assuming no mutation at the modifier locus *M*/*m*:

where  is the mean fitness of the population.
